# Supplementary material for: The impact of girl child marriage on the completion of the first cycle of secondary education in Zimbabwe: A propensity score analysis
Source: PLoS One. 2021 Jun 9;16(6):e0252413. doi: 10.1371/journal.pone.0252413 (PMC8189498; doi:10.1371/journal.pone.0252413)
Supplement: Attachment — Submitted filename: Response to Reviewer_PONErev2.docx [file pone.0252413.s002.docx]

**Response to Reviewers**

**Dear Editor,**

We would like to thank the reviewers for their comments as well as the opportunity to revise our manuscript for further consideration in your journal. We have addressed all the comments from the reviewers and below we provide a detailed description of the revisions made. These changes are also highlighted in yellow in the revised manuscript we submitted.

Reviewer #2: The revised manuscript is much improved with regard to the description of how the outcome variable was measured and the inclusion of a sensitivity analysis. However, the fundamental problems with confounder identification and adjustment remain and must be addressed.

In my initial review I recommended that the authors measure age and age difference variables continuously to improve the accuracy of matching. This was done for the wife’s age but not for the age difference between spouses. I don’t follow the logic of using spousal age difference a categorical variable because there were a large proportion of missing values. Missing values for wife’s age and/or husband’s age would prohibit the estimation of age difference regardless of whether the variable was measured continuously or categorically. Moreover, it is unclear how missing values were handled in the analysis. At a later point in the paper (lines 371-374) the authors state, “The missing indicator method was applied to missing values on covariates such as spousal age difference…” but it is unclear what this means and no citations are provided. If this refers to the use of binary variables to indicate missingness, the authors should acknowledge the high likelihood that this method itself induces bias. (For reference, see Donders et al. Journal of Clinical Epidemiology 2006.)

Response: we have used both age and spousal age difference as continuous variables

In my initial review I raised concerns regarding control for variables that may be affected by the exposure, including household socioeconomic status (SES). The authors assert that it is reasonable to assume that young women marry husbands of the same SES, suggesting that SES is the same prior to and after marriage. This contradicts much of what is known about the relationship between poverty and child marriage. It is also contradicted by the introduction to the paper, which indicates that “…kuzvarira is often a survival tactic where low-income families negotiate with wealthy families to marry off their daughters at a younger age in exchange for grains, cows or money.” This certainly suggests that SES differs prior to and after marriage, especially in the case of child marriage, and should not be adjusted for. For further information on identifying potential confounders, I recommend that the authors consult recent work by VanderWeele (Principles of confounder selection, European Journal of Epidemiology, 2019).

Response: we have removed wealth quintile from the analysis.

In my initial review I asked for clarification regarding whether all women were in their first unions. Given that the DHS only collects information on a woman’s current spouse, women in second or later unions would not have provided relevant information about their first spouse. In response, the authors included a variable for union number in the model. Union number cannot possibly affect age at marriage and therefore cannot be a confounder. It should not be adjusted for. An alternative would be to exclude women who were not in their first unions from the analysis, though this may result in problems with selection bias.

Response: we have excluded women who were not in the first union from the analysis
In the revised version of the manuscript the authors have added age at first sex and age at first birth to their model. While sex and pregnancy may lead to child marriage, for a substantial number of young girls, age at marriage probably determines age at first sex and age at first birth. For girls whose first birth follows their marriage, this variable is almost certainly on the causal pathway between age at marriage and educational attainment. Again, this strongly indicates that these variables should not be treated as confounders. The proportion of girls who report an age at first sex prior to age at first marriage (which can be estimated using DHS data) might guide subsequent analytic decisions.

We have differentiated between women who had sexual debut before marriage and those whose debut was after marriage

On line 463 the authors report the prevalence of child marriage among women between 17 and 24 years of age. This doesn’t correspond with the analytic sample of 20-29-year-olds described in the rest of the paper. Is this a typing error?

We have corrected the error

We have also copy edited the manuscript for grammatical errors.

Sincerely,

Dr Annah Bengesai

University of KwaZulu-Natal

South Africa

Email: [bengesai@ukzn.ac.za](mailto:bengesai@ukzn.ac.za)

ORCID [0000-0002-2711-8530](http://orcid.org/0000-0002-2711-8530)

Dr Lateef A. Babatunde

University of Ilorin, Nigeria

Email: [Amusa.lb@unilorin.edu.ng](mailto:Amusa.lb@unilorin.edu.ng)

And

Dr Felix Makhonye

University of KwaZulu-Natal

Email: MakonyeF@ukzn.ac.za
